# No evidence that male killing (MK) *Wolbachia* in *Drosophila innubila* protects against Drosophila innubila Nudivirus (DiNV) infection

**DOI:** 10.17912/micropub.biology.002246

**Published:** 2026-10-21

**Authors:** Taiye S. Adewumi, Abigail Lewis, Robert L. Unckless

**Affiliations:** 1 Molecular Biosciences, University of Kansas, Lawrence, KS, United States

## Abstract

*Wolbachia* can influence host–pathogen interactions, particularly through its protective effects against RNA viruses in insects. Although prior studies failed to find any evidence for *Wolbachia* protecting against DNA viruses, the viruses utilized in those studies were usually not pathogens found in natural *Drosophila* populations. In this study, we compared survival, viral genome copy number, and infectious-virus production (as measured by flow cytometry after cell culture infection) between W⁺ and W⁻ flies following Drosophila innubila Nudivirus (DiNV) infection. We found no evidence in any of these assays that *Wolbachia* protects D *. innubila* against DiNV.

**Figure 1. Male killing Wolbachia does not protect D. innubila from DiNV:** **A)** Kaplan-Meier survival curve for infection with DiNV. Mated females of either Wolbachia free or male killing Wolbachia *D. innubila* were either mock injected or DiNVinjected and survival was monitored for 14 days. Lines represent the average survival proportion across all replicates shading represents the 95% confidence interval. Cox proportional hazard shows no significant difference (p>0.05) in survival between the two genotypes. **B)** Power analysis plot shows that for a sample size of ~180 flies, there is 97% probability of detecting large survival differences (Hazard ratio > 1.8), 76% probability of detecting moderate survival differences (Hazard ratio > 1.5) and 23% probability of detecting small survival differences (Hazard ratio > 1.2). **C)** Relative viral titre was compared over time between the two genotypes. Relative viral titre was calculated as ΔCq = Cq_tpi − Cq_pif3, where Cq_tpi represents the Cq value of the host reference gene and Cq_pif3 represents the Cq value of the viral target. These ΔCq values are plotted as log₂(relative titre), with higher values indicating greater viral titre. There was no significant genotype effect across days (p > 0.05). Each dot represents one biological sample, consisting of a pool of five flies. **D)** The percentage of *Drosophila virilis* (Dv-1) cells infected was measured 48 hours after inoculation with DiNV-infected or uninfected with *D. innubila* W⁻ and W⁺ flies collected at day 5. Arcsine square root-transformed proportions showed no significant difference in the percentage of infected cells between W⁻ and W⁺ groups (p > 0.05). The y-axis reflects actual percentages of DiNV-positive cells and not transformed proportions from arscine square root. All boxplots show the median, interquartile range, and data spread for each treatment group. The horizontal line within each box represents the median, and the lower and upper box limits represent the 25th and 75th percentiles, respectively. Whiskers extend to the most extreme values within 1.5× the interquartile range, and points beyond the whiskers are shown as outliers.

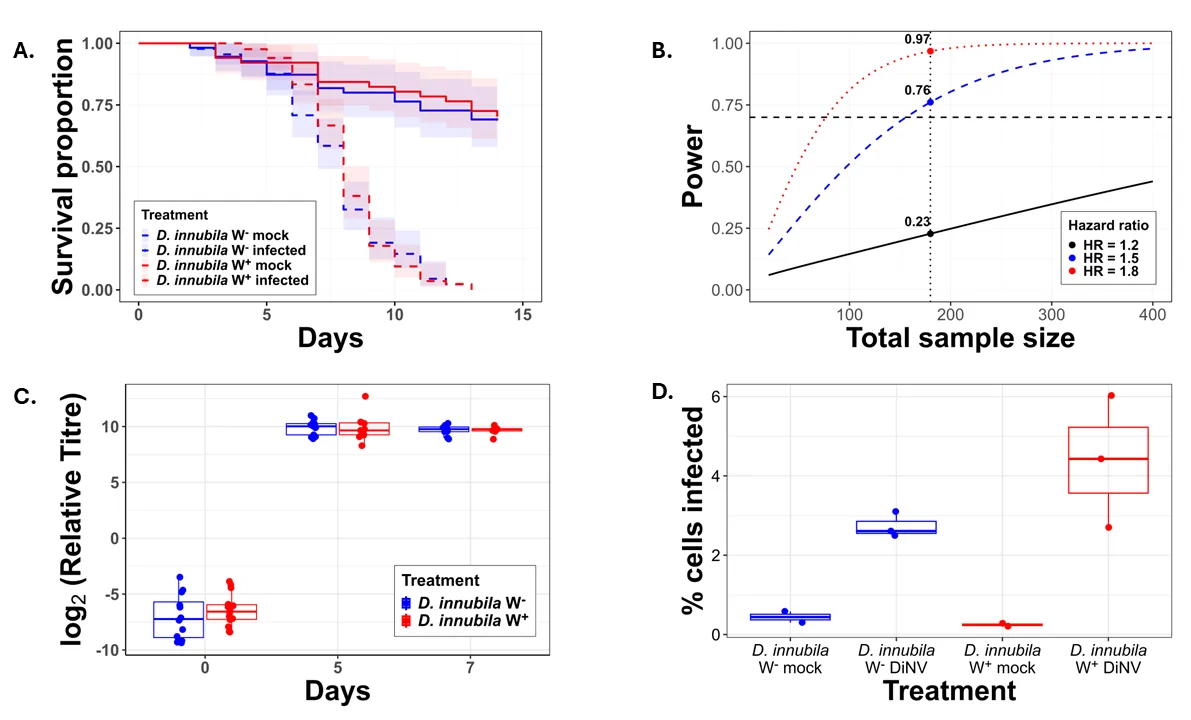



## Description

*Wolbachia* is an obligate endosymbiont that infects more than half of all insect species (Weinert et al., 2015; Zug & Hammerstein, 2012). Over the past 20 years, *Wolbachia* has also been the focus of translational research because it protects insect hosts against RNA viruses, including protecting mosquitoes from Dengue and Zika virus (Lindsey et al., 2018; Mohanty et al., 2016). While less well-studied, *Wolbachia* appears to protect *Drosophila* against a wide range of pathogens: fungi (Perlmutter et al., 2025), several RNA viruses (Cogni et al., 2021; Hedges et al., 2008; Pimentel et al., 2025; Teixeira et al., 2008; Unckless & Jaenike, 2012), parasites (Bian et al., 2013; Hughes et al., 2011; Kambris et al., 2010; Moreira et al., 2009), and bacterial pathogens (Wong et al., 2011; Ye et al., 2013) although the evidence for bacteria is mixed (Rottschaefer & Lazzaro, 2012). Broader *Wolbachia* phenotypes differ among different host groups and *Wolbachia* strains (Wang et al., 2016). There is enormous genetic diversity among strains, with 18 different recognized supergroups (Lefoulon et al., 2020). Because they are maternally transmitted at rates often below 100%, *Wolbachia* is maintained in populations by balancing the fitness benefits to the cytoplasmic lineage with the rate of loss due to imperfect transmission. One of the most striking *Wolbachia-* associated phenotypes is male-killing where infected mothers produce only daughters since males usually die as embryos (Hurst & Jiggins, 2000; Ikeda, 1970; Sugimoto & Ishikawa, 2012; Unckless & Jaenike, 2012). *Drosophila innubila* harbors a male-killing *Wolbachia* at frequencies of about 30% in females (Dyer & Jaenike, 2005; Hill et al., 2022). This particular *Wolbachia* appears to be maintained by both male-killing specific benefits (avoidance of competition with male siblings) and through protection against RNA viruses (though the relative importance of the viral protection in the wild is unknown) (Unckless & Jaenike, 2012).

*D. innubila* is also plagued by a large double stranded DNA virus, the Drosophila innubila Nudivirus (DiNV) (Unckless, 2011). The virus is highly virulent in the native host and is also found at high frequency in natural populations (Hill & Unckless, 2020; Schedl et al., 2026; Unckless, 2011). We recently developed a tractable lab model for DiNV allowing us to perform controlled infection with a viral stock grown in cell culture (Mulkey et al., 2026; Schedl et al., 2026). A similar virus, Kallithea virus, was also discovered in *Drosophila melanogaster* and has been characterized (Palmer et al., 2019; Palmer et al., 2018; Webster et al., 2015).

Teixeira et al *.* (2008 *),* reported that *Wolbachia* in *D. melanogaster* does not protect (in terms of mortality or viral titre) against the DNA virus, Insect Iridescent Virus 6 (IIV-6). Another study showed that *Wolbachia* does not protect *D. melanogaster* from Kallithea virus infection (Palmer et al., 2018). Similarly, Cogni et al., showed that there was no significant difference in the prevalence of Kallithea virus in *Wolbachia* infected versus *Wolbachia* -free wild caught flies (Cogni et al., 2021). Nevertheless, the enormous genetic and phenotypic diversity of *Wolbachia* strains (Lefoulon et al., 2020) suggests that different strains can provide diverse benefits to the host, and *Wolbachia* in *D. innubila* might protect against DiNV. We sought to test whether the male- *killing* *Wolbachia* *in* *D. innubila* protects against DiNV infection by monitoring survival, viral genome copy number and the amount of viable virus recovered from infected flies.

We first compared survival following injection with DiNV (approximately, ~360 FFU per fly) between *Wolbachia* -infected (W⁺) and uninfected (W⁻) female *D. innubila* . Survival following DiNV infection was compared between W⁺ and W⁻ flies using a Cox proportional-hazards model stratified by experimental block, allowing the baseline hazard to vary among the three independent experimental blocks. *Wolbachia* infection status did not significantly affect survival following DiNV infection (p = 0.73; Supplementary Table 1). The proportional-hazards assumption was not significantly violated in the stratified model (global Schoenfeld test, p = 0.11). Thus, after accounting for block-specific variation in baseline mortality risk, we detected no significant difference in survival between W⁺ and W⁻ flies following DiNV infection ( Figure 1A ). This indicates that DiNV infection reduces host survival irrespective of their *Wolbachia* status. We performed a power analysis to determine whether our sample size (n= ~90 per treatment) was sufficient to detect differences based on *Wolbachia* infection status ( Figure 1B ). Our power analysis showed that our sample size was enough to detect moderate (Hazard Ratio > 1.5, meaning that *Wolbachia* uninfected flies are 50% more likely to die at a given timepoint) to high genotype-dependent survival differences nearly 80% of the time. A recent study (Asselin et al., 2026), detected the protective effect of *Wolbachia* on *D. melanogaster* against different titers of Drosophila C virus (DCV) with hazard ratios of 2.01 and 2.82, both exceeding our detectable-effect threshold of hazard ratio > 1.5, indicating that our study was adequately powered to detect *Wolbachia* protection of a similar magnitude. However, smaller protective effects, particularly those that might occur against a DNA virus such as DiNV, cannot be ruled out.

Next, we tested whether *Wolbachia* infection status influenced DiNV genome copy number at three time points after infection using quantitative PCR on DNA copy number. We observed no difference in DiNV genome copy number associated with *Wolbachia* status at days 0, 5, and 7 post infection ( Figure 1C ). Consistent with this, our model, viral copy number ~ *Wolbachia* * day + block, showed no significant *Wolbachia* effect (p=0.72, supplementary table 4), *Wolbachia* * day interaction (p=0.78), or block effect (p=0.27). However, there was a significant effect of day (p < 0.0001), consistent with the virus increasing over the course of the infection. Overall, these results suggest that *Wolbachia* infection status does not affect DiNV genome copy number in *D. innubila* .

Finally, we determined whether *Wolbachia* infection status influences the number of viable DiNV particles. This should be analogous to genome copy number measured by quantitative PCR, but instead of measuring DNA copy number, we count actual infectious particles by inoculating virus isolated from infected whole flies into a cell culture line. Similarly, when we modeled the percentage of cell infected, we found no significant difference (p=0.55) between W⁻ and W⁺ flies, or *Wolbachia* * infection interaction (p=0.125, supplementary table 5) in the percentage of infected cells. As expected, a post hoc analysis showed that DiNV-infected samples were significantly different (Tukey HSD p < 0.05) from their respective mock-inoculated controls ( Figure 1D ). Consistent with the qPCR results showing no detectable difference in viral DNA abundance, the cell-based infectious virion approach also revealed no detectable difference in infectious-virus production between W⁺ and W⁻ flies. Together, these complementary assays provide further evidence that *Wolbachia* infection status did not influence DiNV infection under the conditions tested.

Overall, these results suggest that the male-killing *Wolbachia* strain in *D. innubila* does not provide detectable protection against DiNV under the conditions tested. However, because *Wolbachia* density was not quantified in the experimental flies, we cannot exclude the possibility that variation in symbiont density or tissue distribution may influence its interaction with DiNV.

## Methods

**Fly husbandry and genotypes**

*Drosophila innubila* *Wolbachia* -free flies, described as the “genome” line by Hill et al. (2019), were maintained according to Schedl et al. (2026). Briefly, flies were reared on Carolina 4–24 Instant *Drosophila* Medium (Plain, Item #173200; Carolina Biological Supply Co., NC, USA) supplemented with approximately 1 g of white mushroom ( *Agaricus bisporus* ) and a dental roll. The *Wolbachia* -infected (W⁺) line was maintained under the same conditions and has been continuously backcrossed for approximately 18 months (~20 generations) by crossing W⁺ females to males from the *Wolbachia* -free (W⁻) genome line. This crossing scheme repeatedly introgressed the W⁺ nuclear background into that of the W⁻ genome line and was therefore expected to produce highly similar nuclear genetic backgrounds between the two lines. The W⁻ genome line was previously used for the *D. innubila* reference genome and showed no evidence of residual heterozygosity.

*Wolbachia* status was monitored throughout laboratory maintenance using the characteristic male-killing phenotype of infected *D. innubila* . W⁺ females consistently produced strongly female-biased offspring, whereas the W⁻ genome line produced both male and female offspring. The association between *Wolbachia* infection and male killing in *D. innubila* has previously been established molecularly, including detection of *Wolbachia* using the *wsp* gene and an association between *Wolbachia* density, male-killing efficacy, and maternal transmission (Unckless et al., 2009). All fly lines were maintained under a 12 h light:12 h dark cycle at 20 °C.

**Source of virus**

We aimed to create a clonal-derived virus for infections. To do so, the initial virus was recovered from wild caught *D. innubila* flies homogenized in 100ul of viral buffer (Unckless, 2011), the homogenate was subsequently passed in *Drosophila innubila* cell line (Dinn-1) to produce DiNV ^2^ (the second passage after the initial infection of Dinn-1 primary cells) (Schedl et al., 2026). DiNV ^2^ was diluted at 1:10, 1:100 and 1:1000 and inoculated on Dinn-1 cells with 16 biological replicates for each dilution. DNA was extracted from each sample, and PCR was used to amplify DiNV p47 gene. A PCR positive sample was detected at 1:1000 dilution and was expanded in Dinn-1 cells to generate a large virus stock. The resulting virus stock was sequenced to check for residual heterozygosity and named DiNV **^cl^** as sequencing results indicated that most previously segregating sites had been fixed.

**Infection methods for flies**

Five-eight day old mated female flies were injected using a Nanoject II® from Drummond Scientific Company (PA, USA) according to methods described by (Schedl et al., 2026). DiNV **^cl^** was injected into every fly at 27.6nL (1.31 x 10 ^7^ FFU/mL, ~360FFU per fly). Survival was counted daily and flies were tipped to new food every two days until experiments ended (all virus-infected flies died).

**Viral genome copy number determination**

*D. innubila* (W **^-^** and W **^+^** ) were injected as described above and frozen at days 0 (immediately after injection), 5 and 7. For each treatment and time point, the experiment was conducted in three independent experimental blocks. Five biological replicates were included per treatment and time point in Blocks 1 and 2, and four biological replicates were included in Block 3, resulting in a total of 14 biological replicates per treatment per time point. DNA was extracted from frozen flies using Qiagen Puregene kit in a modified protocol described in (Schedl et al., 2026). qPCR (CFX Connect Real Time PCR System and the Bio-Rad CFX Manager 3.1, Bio-Rad, Hercules, CA, USA) was used to determine viral genome copy number as described in (Mulkey et al., 2026; Schedl et al., 2026), using *tpi* primers as the housekeeping for *D. innubila* and *pif3* as target gene for DiNV. In brief, each reaction included one μL of template DNA, five μL of SsoAdvanced Universal SYBR Green Supermix (BioRad 1725270), 0.5 μL of 10 μM forward primer, 0.5 μL of 10 μM reverse primer, and three μL of molecular grade water. The qPCR program used included: An initial denaturation step of 3 min at 95 °C, followed by 40 cycles of 10 s at 95 °C and 30 s at 60 °C, followed by a 5 min 95 °C extension. This is then followed by a melt curve section that increases by 0.05 °C every 5 s from 55 °C to 95 °C.

**Flow cytometry-based detection of DiNV-infected genotypes in Dv-1 cells**

*Drosophila virilis* (Dv-1) cells were grown in cell culture according to methods described by (Mulkey et al., 2026). *Wolbachia* -free (W⁻) and *Wolbachia* -infected (W⁺) flies, either uninfected or collected at 5 dpi following DiNV infection, were homogenized in PBS in pools of five flies. For the infected groups, three independent biological replicates were analyzed per genotype (W⁺ and W⁻), with each replicate consisting of a pool of five flies collected at 5 dpi. Each pool was independently homogenized, inoculated into a single 48-well of Drosophila virilis (Dv-1) cells, and analyzed by flow cytometry. The percentage of DiNV-positive cells was determined for each infected biological replicate. Anti-capsid (vp39) antibodies were used for detection of DiNV (Schedl et al., 2026). Briefly, Cells were pelleted at 1500 rpm for 5 min, fixed and permeabilized in chilled 80% acetone at −20°C for 30 min and washed four times with cold 1× PBS. Cells were then blocked in 2% BSA for 1 hour at room temperature, followed by incubation with affinity-purified anti-p39 primary antibody diluted 1:2000 in 2% BSA overnight at 4°C. After three PBS washes, cells were incubated with Alexa Fluor 488 goat anti-rabbit secondary antibody diluted 1:4000 in 2% BSA for 1 h at room temperature in the dark. Cells were washed three times with cold PBS, resuspended in 500 µL of 1× PBS. Mock or virus infected cells were analyzed by flow cytometry using an Agilent NovoCyte Advanteon 3L flow cytometer (V8-Y6-B7 configuration, 23 detectors, equipped with 405, 488, and 640 nm lasers), using the NovoExpress Software (version 1.6.2). DiNV-positive cells were identified based on green fluorescence following excitation with the 488 nm laser. For each sample, 20,000 cellular events were acquired by flow cytometry. Cells were first identified based on forward- and side-scatter characteristics to exclude debris. DiNV-positive cells were defined as events with green fluorescence above the threshold established using uninfected W⁺ and W⁻ samples as negative controls. Two independent pools of uninfected flies were analyzed per genotype for the purpose of establishing the fluorescence-gating threshold and were not treated as experimental comparison groups. The percentage of DiNV-positive cells was calculated relative to the gated cellular population for each sample. Representative flow-cytometry plots illustrating the gating strategy and separation of DiNV-positive and negative populations are provided in Supplementary Figures 1 and 2.

**Statistical analysis**

All statistical analyses were performed in R (R Core Team, 2024). For the survival experiment, daily mortality data were converted to individual-level survival data using a custom R function, with survival time represented as the number of days from injection to death or the end of the observation period. Survival analyses were performed using the survival package, and Kaplan–Meier curves were generated using the survminer package (Kassambara et al., 2024; Therneau & Grambsch, 2000).

The experiment consisted of three independent experimental blocks, with a total of 89 W⁻-DiNV, 55 W⁻-mock, 84 W⁺-DiNV, and 51 W⁺-mock flies analyzed across all blocks (n = 279). Flies remaining alive at the end of the observation period were treated as right-censored in the Kaplan–Meier analysis.

To test whether *Wolbachia* infection status affected survival following DiNV infection, the statistical analysis was restricted to DiNV-infected flies (89 W⁻-DiNV and 84 W⁺-DiNV; n = 173). Survival was analyzed using a Cox proportional-hazards model stratified by experimental block: Surv(time-to-death, status) ~ *Wolbachia* + strata(Block)

Experimental block was included as a stratification variable to allow the baseline hazard function to differ among the three independent experimental blocks while estimating a common effect of *Wolbachia* genotype across blocks. The proportional-hazards assumption for the final model was evaluated using Schoenfeld residuals and was not significantly violated (global test, p = 0.11).

The power analysis was performed in R using the powerSurvEpi package (Qui et al., 2025) to estimate the sample size required to detect differences in survival between treatment groups under different hazard ratio assumptions. Power curves were generated by plotting estimated power across a range of total sample sizes, with the current sample size indicated on the plot.

Viral genome copy number was quantified by qPCR and analyzed using linear models in R. Relative viral genome copy number was calculated from qPCR Cq values as ΔCq = Cq_tpi − Cq_pif3, where Cq_tpi represents the Cq value of the host reference gene and Cq_pif3 represents the Cq value of the DiNV target gene. Treatment effects across time points were tested using models that included treatment/genotype, day, their interaction, and block where appropriate. Primers and qPCR protocols including reagents were performed according to (Mulkey et al., 2026)

For the flow-cytometry assay, the percentage of DiNV-positive cells was calculated independently for each biological replicate. Proportional infection data were arcsine-square-root transformed prior to statistical analysis. Differences between infected W⁺ and W⁻ groups, each represented by three biological replicates, were evaluated using ANOVA, followed by Tukey HSD post-hoc comparisons where appropriate. The uninfected samples were used to establish fluorescence-gating thresholds and were not included as experimental treatment groups in the primary comparison of infectious-virus production. Given the limited number of biological replicates, nonsignificant differences were interpreted as a lack of detectable difference under the conditions tested. Plots were generated in R using ggplot2 (Wickham, 2016).

## Reagents

**Table:** 

Fly rearing	Source
Instant Drosophila media	Carolina Biological Supply Co. Item: 173200, NC, USA

**Table:** 

Antibody	Animal and clonality	Description
Anti-capsid vp39 antibody	Rabbit Polyclonal	Pacific Immunology (Ramona, CA). A peptide sequence (DGRREIISNSNDQTFRPIHRPVIC) matching a viable antigenic target on the VP39 protein was synthesized.
